# Trends in Usage and Outcomes for Expanded Criteria Donor Kidney Transplantation in the United States Characterized by Kidney Donor Profile Index

**DOI:** 10.7759/cureus.887

**Published:** 2016-11-22

**Authors:** Aparna Rege, Bill Irish, Anthony Castleberry, Deepak Vikraman, Scott Sanoff, Kadiyala Ravindra, Bradley Collins, Debra Sudan

**Affiliations:** 1 Surgery, Duke University Medical Center; 2 Health Outcomes Research & Biostatistics, CTI Clinical Trial and Consulting; 3 Division of Abdominal Transplantation, Duke University Medical Center; 4 Transplant Nephrology, Duke University Medical Center

**Keywords:** kidney transplant, deceased donor kidneys, expanded criteria donor, clinical outcomes, kidney graft survival, post-transplant mortality, kdri: kidney donor risk index, kdpi: kidney donor profile index, standard criteria donor

## Abstract

There has been increasing concern in the kidney transplant community about the declining use of expanded criteria donors (ECD) despite improvement in survival and quality of life. The recent introduction of the Kidney Donor Profile Index (KDPI), which provides a more granular characterization of donor quality, was expected to increase utilization of marginal kidneys and decrease the discard rates. However, trends and practice patterns of ECD kidney utilization on a national level based on donor organ quality as per KDPI are not well known. We, therefore, performed a trend analysis of all ECD recipients in the United Network for Organ Sharing (UNOS) registry between 2002 and 2012, after calculating the corresponding KDPI, to enable understanding the trends of usage and outcomes based on the KDPI characterization. High-risk recipient characteristics (diabetes, body mass index ≥30 kg/m^2^, hypertension, and age ≥60 years) increased over the period of the study (trend test p<0.001 for all). The proportion of ECD transplants increased from 18% in 2003 to a peak of 20.4% in 2008 and then declined thereafter to 17.3% in 2012. Using the KDPI >85% definition, the proportion increased from 9.4% in 2003 to a peak of 12.1% in 2008 and declined to 9.7% in 2012. Overall, although this represents a significant utilization of kidneys with KDPI >85% over time (p<0.001), recent years have seen a decline in usage, probably related to regulations imposed by Centers for Medicare & Medicaid Services (CMS).

When comparing the hazards of graft failure by KDPI, ECD kidneys with KDPI >85% have a slightly lower risk of graft failure compared to standard criteria donor (SCD) kidneys with KDPI >85%, with a hazard ratio (HR) of 0.95, a confidence interval (CI) of 0.94-0.96, and statistical significance of p<0.001. This indicates that some SCD kidneys may actually have a lower estimated quality, with a higher Kidney Donor Risk Index (KDRI), than some ECDs. The incidence of delayed graft function (DGF) in ECD recipients has significantly decreased over time from 35.2% in 2003 to 29.6% in 2011 (p=0.007), probably related to better understanding of the donor risk profile along with increased use of hypothermic machine perfusion and pretransplant biopsy to aid in optimal allograft selection.

The recent decline in transplantation of KDPI >85% kidneys probably reflects risk-averse transplant center behavior. Whether discard of discordant SCD kidneys with KDPI >85% has contributed to this decline remains to be studied.

## Introduction

The existing donor supply of kidney allografts is unable to meet the growing demand of patients awaiting transplantation. Since 2002 the number of candidates on the kidney transplant waitlist has nearly doubled from just over 50,000 to more than 96,000 by 2013. Although more than 15,000 patients on the waiting list underwent a kidney transplant in 2013 in the United States (US), more than 7,000 candidates were removed from the waiting list due to death or becoming too sick to undergo transplant [1]. To address organ shortages, a policy enabling the use of ECD kidneys (donor age ≥60 years or 50 to 59 years with at least two of the following: history of hypertension, serum creatinine level >1.5 mg/dL, or cerebrovascular cause of death) was implemented in November 2000 [2] with the concept that, although there would be a 70% greater likelihood of graft loss when compared to SCD kidneys, the survival benefit compared to dialysis would still be significant [3]. Survival and improvement in quality of life with ECD kidney transplantation has been demonstrated previously [4-6]; however, the patterns of ECD use on a national level are not well delineated, and there is increasing concern in the transplant community that ECD utilization practices are declining, thereby limiting access to life-saving transplants among viable candidates [7].

In 2012 the Organ Procurement and Transplantation Network (OPTN) introduced KDPI, which compiles 10 donor factors (instead of four in the ECD definition) that are independently associated with all-cause allograft survival associated with the use of that particular organ. A donor KDPI >85% (DK85) is thought to be equivalent to an ECD kidney. KDPI was the basis of the new allocation policy introduced toward the end of 2014 to increase utilization of marginal kidneys and decrease the discard rates that would be expected using the older ECD classification [8].

Trends in utilization and outcomes of donor kidneys using KDPI, which provides for more granular characterization of donor quality, have not previously been well studied. Using data from the UNOS national kidney transplant registry, the goals of our study were to (1) assess trends and practice patterns of ECD kidney utilization on a national level based on donor organ quality as per KDPI and (2) reclassification of the trends in postoperative outcomes following ECD kidney transplantation over the past decade over three distinct eras, using the KPDI tool. This analysis included both the ECD and the KDPI classifications because KDPI offers a more granular characterization of donor quality and when ECD kidneys are split by KDPI, some will fall under KDPI <85% with better allograft outcomes compared to KDPI >85%. Kidneys with KDPI >85% are frequently discarded; having some knowledge about the trends of usage and outcome patterns with such kidneys may help decision-making to possibly preserve this valuable organ resource.

## Materials and methods

### Data source

The UNOS Standard Transplant Analysis and Research files were used for this analysis, which contains data regarding every organ donation and transplant event occurring in the United States since October 1, 1987 [1]. Data are compiled from individual centers and entered by trained data entry personnel with quality assurance controls in place including electronic data validation systems and on-site audits of participating institutions. The dataset used for the current study comprises a prospectively collected open cohort of kidney transplantations performed between January 1, 2000, through December 31, 2012. This dataset was used by us for a similar analysis of ECD kidneys when the prior dichotomous kidney classification system (i.e., SCD and ECD) was in use. We decided to use this same dataset to further characterize ECD kidneys after calculating the KDPI and analyzing based on the KDPI definition.

### Study population

All adult recipients (age ≥ 18 years) of a deceased donor, kidney-only transplant between January 1, 2000, and December 31, 2012, were eligible for the study. Pediatric kidney transplant recipients, multi-organ transplants, living donor kidney transplants, and recipients with a prior kidney transplant were excluded. The study period was split into three eras: 2000-2002, 2003-2006, and 2007-2012. These eras were chosen to reflect changes in the allocation policy incorporating ECD kidneys that were implemented in 2002 [2] and the CMS program evaluation initiative in 2007 [9]. Trend and outcomes analyses were restricted to recipients of ECD kidneys only after calculating the KDPI; therefore, no comparative analyses to recipients of SCD kidneys were performed.

### Follow-up

Outcome data for each patient were ascertained from the date of kidney transplantation until graft failure (which includes death, retransplantation, or return to permanent dialysis) or the end of the study period (December 31, 2012).

### Variable definitions

Risk Factors

Patient outcomes can be impacted by a variety of transplant-related [10-13], donor [11-12], and recipient factors [13-14]. The UNOS database collects data on several of these factors both pre- and post-transplantation. Risk factors evaluated for inclusion in risk-adjusted multivariable analyses included:

1. Donor characteristics: Age, diabetes, hypertension, terminal serum creatinine level (mg/dL), body mass index (BMI) ≥35 kg/m^2^, the cause of death (cerebrovascular versus other), ECD, DGF, donation after cardiac death status, and KDRI [14]. Based on the KDRI, the KDPI is calculated.

2. Recipient characteristics: Age, sex, race/ethnicity, comorbidities [diabetes, hypertension, chronic obstructive pulmonary disease (COPD), cerebrovascular disease], BMI (kg/m^2^), dialysis requirement at the time of transplant (yes/no), and days on the waitlist.

3. Transplant-related characteristics: Cold ischemia time (CIT) in hours, hypothermic machine perfusion [15] of donor allografts (yes/no), pretransplant biopsy performed (yes/no), peak panel reactive antibody (grouped as <20%, 20-49%, 50-79%, ≥80%).

The proportion of missing data for the KDRI (60% of records), recipient cerebrovascular disease (36% of records), and recipient hypertension (35% of records) precluded the use of these parameters in the multivariable analyses.

### Endpoints

Endpoints for analysis included:

-KDPI >85% utilization rate as a proportion of all cadaveric kidney transplantations. -DGF defined as the need for dialysis within the first seven days post transplantation. -All-cause graft failure defined as patient death or graft loss (i.e., first retransplantation, graft nephrectomy, or resumption of chronic dialysis), whichever came first for any reason at any time during follow-up. -Graft survival calculated from the date of transplantation until the date of graft failure. Patients alive with a functioning graft were censored at the date of last known follow-up. -Overall patient survival calculated from the date of transplantation until the date of patient death. Patients alive were right censored at the date of last known follow-up.

### Statistical analysis

Baseline characteristics were described for the study population stratified by the year of transplantation. Normally distributed continuous data are presented as mean and standard deviation (SD), with median and interquartile range (IQR) for non-normal distributions. Categorical data are presented as counts and percentages. The Cochran-Armitage trend test was used to assess the presence of an association between binary categorical variables (e.g., gender or yes/no for ECD) and year of transplantation. Trends in continuous variables were assessed using ordinary least squares linear regression model; for categorical variables defined in more than two categories (e.g., race), a multinomial regression model was utilized.

Multivariable binary logistic regression modeling was used to assess the simultaneous effect of year of transplantation on the risk of DGF while adjusting for donor-, recipient- and transplant-related risk factors. Odds ratio (OR) and 95% CI were calculated as measures of strength of association and precision, respectively.

Graft and patient survival rates were estimated using the Kaplan-Meier method and compared between years by the log-rank test. Multivariable Cox proportional hazards modeling was used to assess the simultaneous effect of year of transplantation on the risk of graft failure, and separately for risk of patient death while adjusting for donor-, recipient-, and transplant-related risk factors. HR and 95% CI were calculated as measures of strength of association and precision, respectively. 

**Figure 1:**
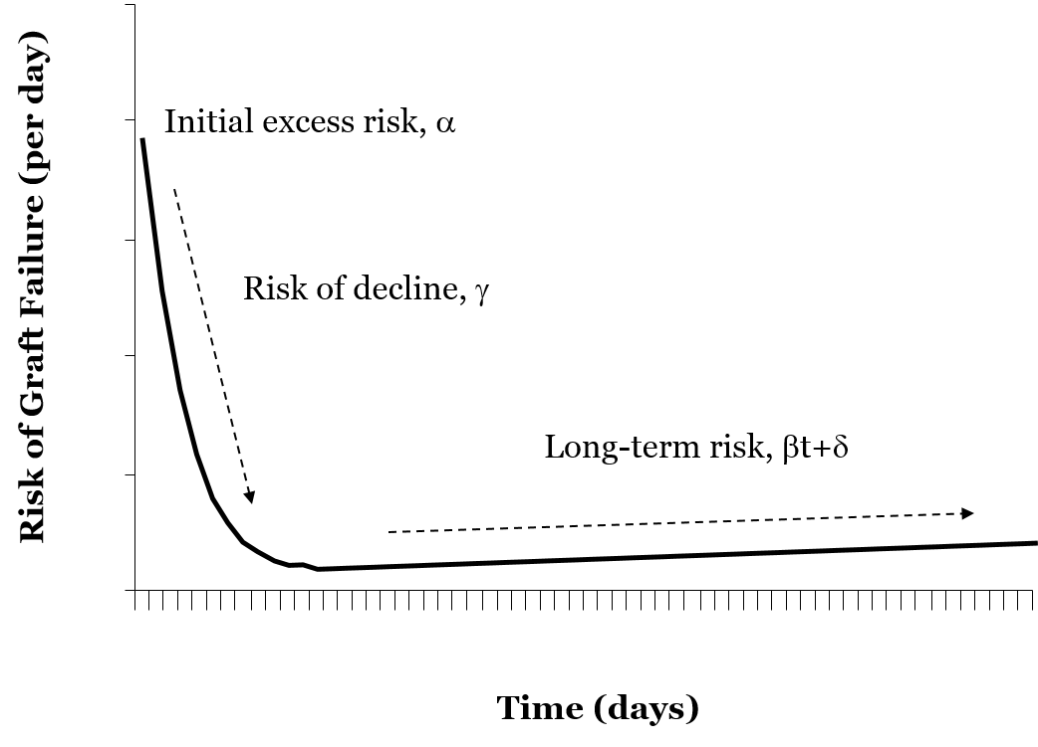
Modified Bailey-Makeham model for risk of graft failure Formula: Risk(t) = αe^-gt ^(short-term) + βt + δ (long term). The y-intercept signifies inital excess risk "α," the vertical slope is indicative of risk of decline "γ," and the horizontal slope implies long-term risk of graft failure βt + δ.

A modified Bailey-Makeham model for risk of graft failure was used to describe rates of graft loss from the time of transplant. We used the graphical representation of the modified model which is a parametric model based on work published in *Transplantation* in 1977 [16]. This model can provide a smooth, continuous estimate of the risk of graft failure post-transplantation, allowing for evaluation of the effect of donor and recipient characteristics on the risk of graft failure; it also permits separate evaluations of short-term and long-term prognosis. The model consists essentially of two parts: Bt+δ, which incorporates parameters to capture the linear increase in the long-term risk of graft failure, coupled with a second part that incorporates parameters "a" and "g" for the short-term risk of graft failure. The parameter "a" defines the initial excess risk of graft failure post-transplant, which diminishes over time with rate constant "g" (Figure 1).

## Results

### Study sample characteristics

The study population includes all adult recipients (age ≥ 18 years) of a deceased donor, kidney-only transplant between January 1, 2000, and December 31, 2012. All living donor transplant recipients, recipients of pediatric kidneys, multi-organ transplants, or recipients with a prior kidney transplant were excluded from the study. Study period includes three different eras: Era One, 2000-2002; Era Two, 2003-2006, which reflects changes in the allocation policy with the implementation of ECD kidneys in 2002; and Era Three, 2007-2012, which reflects implementation of the CMS program evaluation initiative in 2007.

### Donor characteristics

Several donor characteristics were significantly associated with the eras of transplantation as seen in Table 1.* *The use of ECD kidneys with KDPI >85% has significantly decreased over the years in comparison to the use of ECD kidneys with KDPI <85%, with 89.1% in Era One compared to 84.7% in Era Three. The proportion of donors with a history of diabetes and hypertension has increased between the eras (p<0.0001). Donor BMI has increased across the eras; in Era Three, 15.6% of donors had BMI >35 compared to 12.6% in Era One. Use of donor after cardiac death (DCD) kidneys significantly increased in both the KDPI groups across all three eras.

**Table 1:** Donor characteristics The use of ECD kidneys with KDPI >85% has significantly decreased over the years from 89.1% in Era One to 84.7% in Era Three. The proportion of donors with a history of diabetes, hypertension, and higher BMI has increased across the eras (p<0.0001). CVA = cerebrovascular accident.

	Era One: 2000-2002		Era Two: 2003-2006		Era Three: 2007-2012		P value*
Donor Characteristics	KDPI <85%	KDPI >85%	KDPI<85%	KDPI>85%	KDPI <=85%	KDPI >85%	KDPI>85%
Mean Age (SD)	34.8 (16.0)	61.2 (11.8)	36.2 (15.6)	61.4 (10.0)	37.0 (15.4)	59.1 (13.0)	<.0001
% Diabetes	3.0	15.2	4.1	21.5	5.5	26.9	<.0001
% Hypertension	15.8	65.5	20.6	74.1	23.8	77.2	<.0001
% Terminal Creatinine >1.5 mg/dL	11.1	18.1	12.3	17.0	15.6	19.7	.032
Mean Donor BMI (kg/m^2^)	25.8 (22.5)	27.5 (8.1)	26.4 (7.2)	28.0 (12.2)	27.2 (6.7)	28.5 (7.3)	.0006
% Donor BMI ≥35 kg/m^2^	6.9	12.6	8.7	12.3	11.7	15.6	<.0001
% CVA Cause of Death	37.1	84.3	35.9	83.2	31.3	78.1	<.0001
% ECD	9.4	89.1	10.5	88.5	11.0	84.7	<.0001
% DGF	24.3	36.6	25.6	34.1	26.6	34.6	.255
% DCD	3.0	2.7	7.4	6.8	15.0	9.4	<.0001
Mean KDRI (SD)	0.93 (0.24)	1.71 (0.21)	0.95 (0.24)	1.74 (0.23)	0.97 (0.24)	1.74 (0.23)	.0009

**Figure 2:**
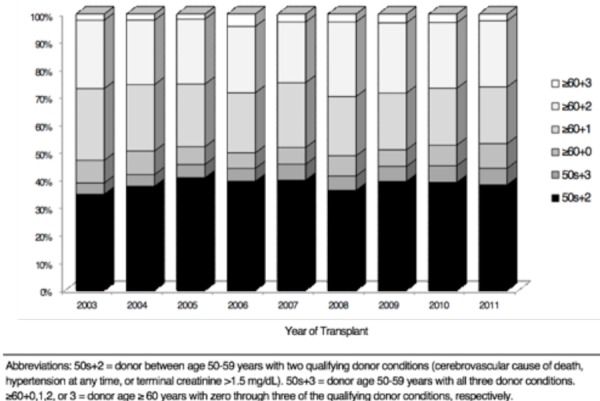
Trend in donor characteristics by the ECD definition

Trends in donor characteristics that defined the allografts as ECD are provided in Figure 2. For all years, the majority of ECD kidneys were from donors age 50-59 with two qualifying comorbidities, ranging from 34.4% to 39.9% of all ECD kidneys transplanted. The next most common donor profile was age ≥60 with two qualifying comorbidities, ranging from 21.4% to 26.6% of ECD kidneys transplanted. This was followed by age ≥60 with only one qualifying comorbidity, ranging from 20.1% to 25.7% of ECD kidneys transplanted. The proportion of donors aged 50-59 with three qualifying conditions significantly increased over time (p=0.008), while the proportion of donors age ≥60 with one qualifying comorbidity significantly decreased (p<0.001).

### Recipient characteristics

Recipients of kidneys with KDPI >85% have become increasingly older over time, as seen in Table 2. The mean (standard deviation) age of recipients in Era Three was 60.9 (10.3) years compared to 55.6 (12.3) in Era One (p<0.001). Moreover, the proportion of patients older than 60 years of age increased from 39.3% in Era One to 56.9% in Era Three (p<0.001). The proportion of recipients with diabetes has significantly increased over time 34.3% in Era One vs. 47.8% in Era Three, p<0.001), as did the proportion of recipients with a BMI >35 km/m^2^ increasing from 7.0 % in Era One to 10.3% in Era Three (p<0.001). 

**Table 2:** Recipient characteristics Recipients of kidney with KDPI >85% have become increasingly older over the eras, with higher proportion of recipients >60 years of age (p>0.001). The proportion of recipients with diabetes and BMI >35 has also significantly increased over time (p>0.001).

	Era One: 2000-2002		Era Two: 2003-2006		Era Three: 2007-2012		P value*
Recipient Characteristics	KDPI ≤85%	KDPI >85%	KDPI ≤85%	KDPI >85%	KDPI ≤85%	KDPI >85%	KDPI >85%
Mean Age (SD)	49.0 (12.8)	55.6 (12.3)	50.8 (12.9)	59.3 (10.6)	52.5 (12.9)	60.9 (10.3)	<.0001
% Age >60	20.1	39.3	24.7	50.4	30.0	56.9	<.0001
% Female Gender	39.3	41.1	38.5	36.8	38.9	35.8	<.0001
% Black	30.5	35.6	31.5	36.5	34.7	37.9	.071
% Diabetes	29.0	34.3	32.9	42.2	37.2	47.8	<.0001
Mean BMI (SD) kg/m2	27.0 (5.4)	26.9 (5.2)	27.6 (5.4)	27.4 (5.1)	28.4 (5.5)	28.1 (5.1)	.936
% BMI ≥35 kg/m2	8.1	7.0	9.7	8.0	12.9	10.3	<.0001

### Pretransplant management characteristics

Donor kidney preservation techniques have changed over time. The use of hypothermic machine perfusion for the preservation of kidneys with KDPI > 85% increased from 56% in Era Two to 62.3% in Era Three (p<0.001). Kidneys with KDPI < 85% also saw an increased use of machine perfusion from 33.6% to 40.8%. The proportion of pretransplant biopsies increased from 83.9% in 2003 to 90.2% in 2011 (p<0.001). The mean CIT and % CIT >24 hours were not significantly different between the three eras. Median recipient wait time increased 35% from 679 days in 2003 to 915 days in 2011 (p<0.001). Table 3 below depicts the pretransplant management characteristics.

**Table 3:** Pretransplant management characteristics The use of hypothermic machine perfusion for preservation of kidneys with KDPI >85% increased from 56% in Era Two to 62.3% in Era Three (p<0.001).

	Era One: 2000-2002		Era Two: 2003-2006		Era Three: 2007-2012		P value*
Transplant-related	KDPI ≤85%	KDPI >85%	KDPI ≤85%	KDP I>85%	KDPI ≤85%	KDPI >85%	KDPI >85%
% PRA >20%	16.1	13.6	18.5	13.6	20.9	13.9	.713
Mean CIT (SD) hours	19.1 (8.4)	20.7 (8.7)	18.3 (8.4)	20.0 (9.1)	17.9 (9.8)	19.9 (10.6)	.018
% CIT >24 hours	20.6	25.0	18.9	24.6	18.5	25.1	.784
% Pump	-	-	33.6	56.0	40.8	62.3	<.0001

### Endpoints

**Figure 3:**
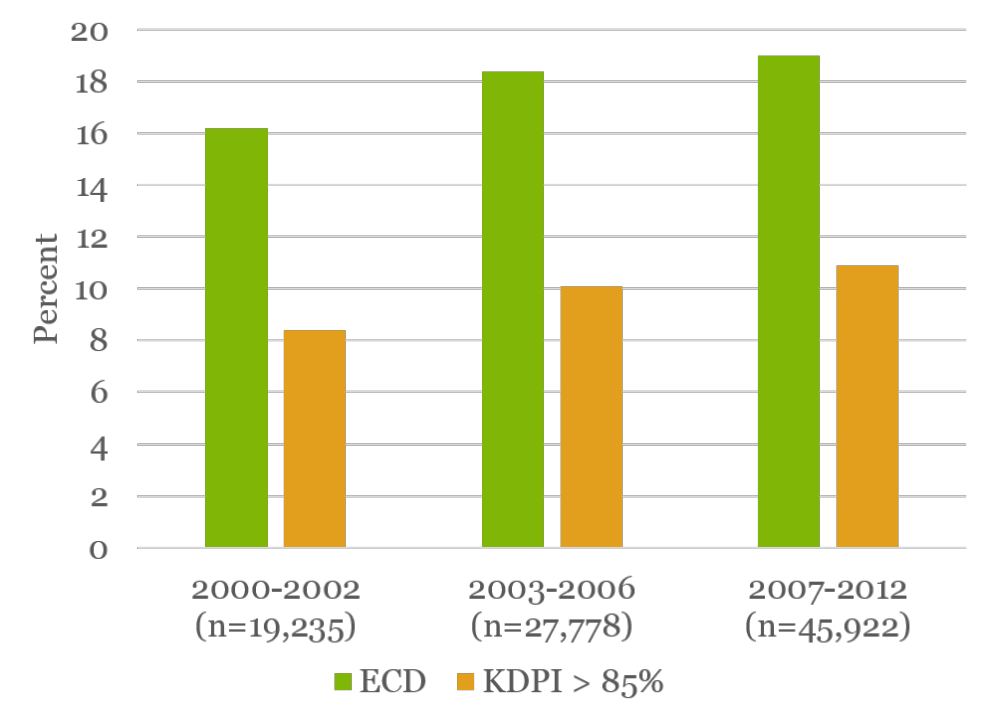
Trend in utilization of ECD and KDPI >85% kidneys across the eras Rates of use of ECD and KDPI >85% kidneys increased during the time period of interest.

The rates of use of lower-quality kidneys were examined by era, taking into consideration both ECD and KDPI >85% designations. As seen in Figure 3 below, the rates of use of ECD and KDPI >85% kidneys increased during the time period of interest. With the newer KDPI designation, some of the ECD kidneys fall into the KDPI <85% category; thus the KDPI >85% incorporates fewer organs.

**Figure 4:**
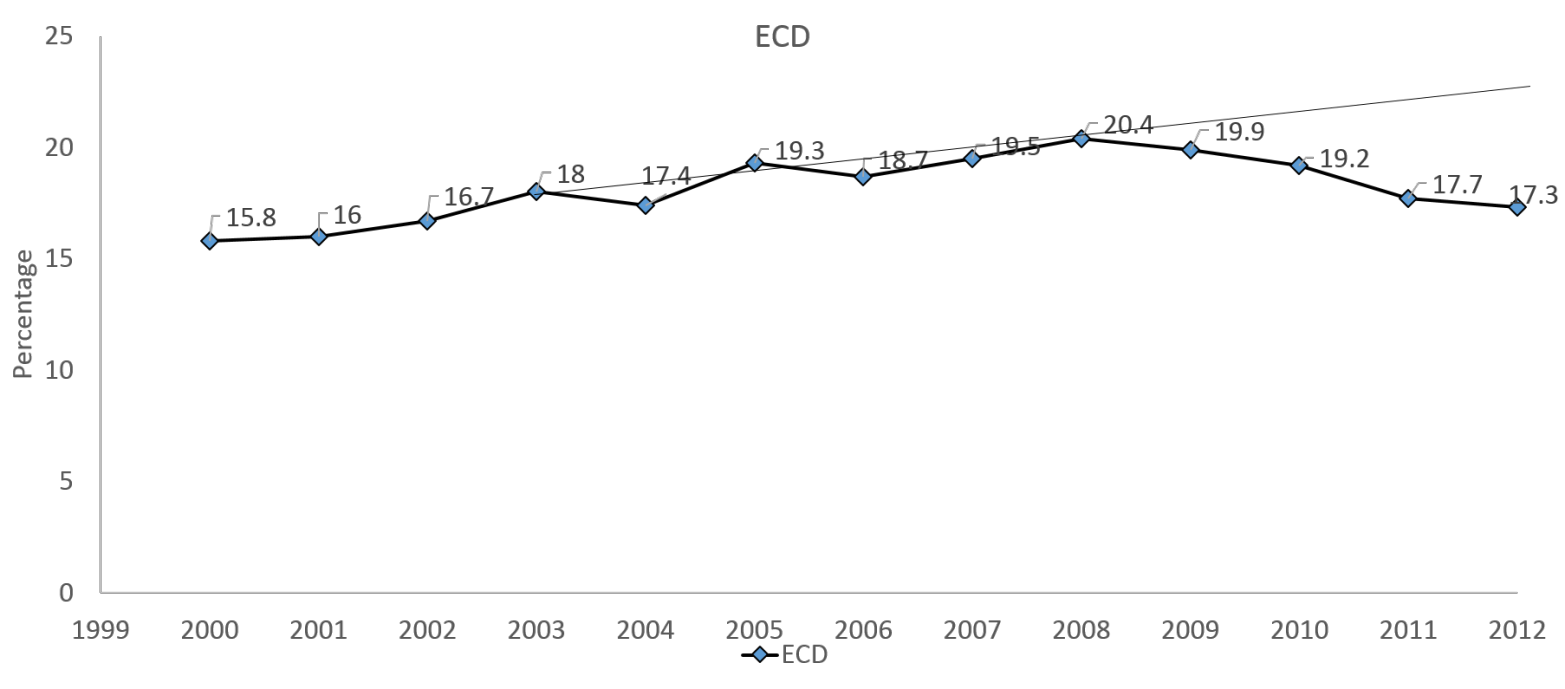
Projected linear regression trend line for ECD kidney utilization Trend line 2003-2008; y=0.0075x + 0.17. A linear regression trend line between 2003 and 2008 estimates the rate of ECD utilization to be around 23.8% in 2012. On the contrary, the proportion of ECD transplants has declined to 17.3% in 2012.

Using time series analysis, the change in the trend of ECD utilization before and after 2008 was significant (p<0.001). To better characterize this decline, when a linear regression trend line was derived between the years of 2003 and 2008, the projected value of ECD kidney use was estimated at 23.8%. However, the actual 2011 ECD utilization rate was down to 17.7%, representing an 18% decline from the projected value of 23.8%, as seen in* *Figure 4.

**Figure 5:**
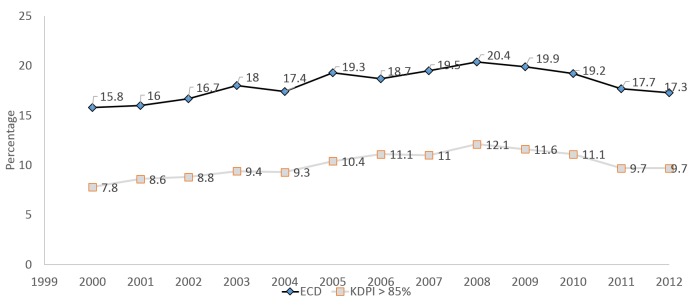
Trend of the use of kidneys by ECD and KDPI classification The use of kidneys with KDPI >85% has declined in the most recent era (2007-2012), despite the upward trend in use in the previous two eras (2000-2002 and 2003-2006).

The proportion of ECD transplants has increased from 18% in 2003 to a peak of 20.4% in 2008, and then declined thereafter to 17.3% in 2012.  In terms of KDPI >85%, transplants increased from 9.4% in 2003 to a peak of 12.1% in 2008 and declined to 9.7% in 2012 (Figure 5). Although there has been a significant use of ECD kidneys since 2000 (p<0.001), the drop in use following 2008 probably suggests CMS regulatory requirements impacting the continued expansion of the use of these kidneys and creating a risk-aversive behavior for transplant centers.

**Figure 6:**
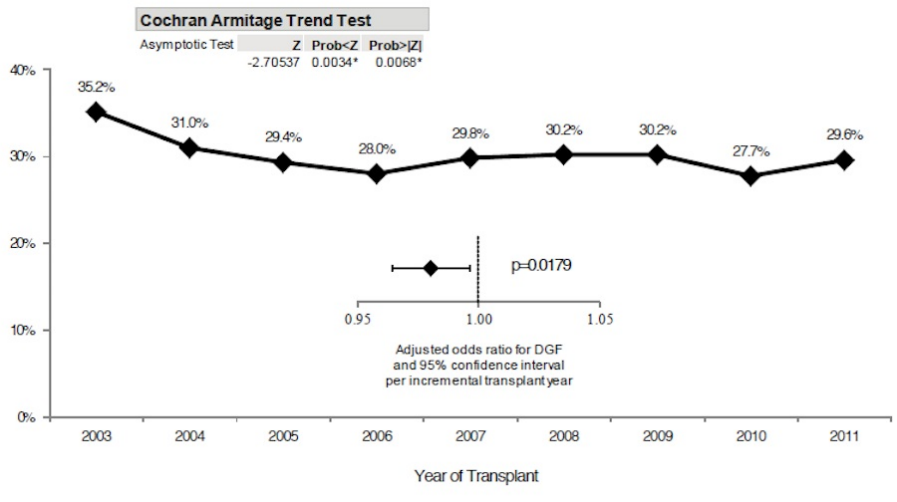
Percentage of ECD kidney transplants with DGF by year of transplant Incidence of DGF has significantly decreased from 35.2% in 2003 to 27.7% in 2010, with a slight increase to 29.6% in 2011 (p=0.007). On multivariable logistic regression, the odds of DGF have significantly decreased per incremental year of transplantation (OR: 0.98; CI: 0.96–0.997; p=0.018).

Over the years, the incidence of DGF in ECD recipients has significantly decreased from 35.2% in 2003 to a low of 27.7% in 2010 and 29.6% in 2011 (p=0.007) as shown in Figure 6. Upon multivariable logistic regression adjusting for potential confounders, the odds of DGF significantly decreased per incremental year of transplantation (adjusted odds ratio per one-year increase: 0.98, CI of 0.96 – 0.997, and p=0.018). 

**Figure 7:**
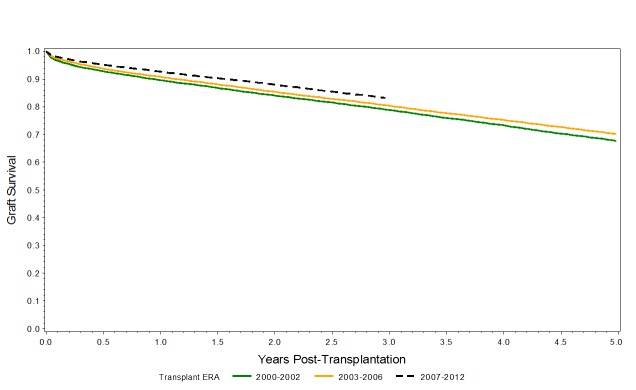
Kaplan-Meier graft survival curves for the entire cohort: ECD, KDPI 85% Kaplan-Meier analysis demonstrates the improvements in graft survival for all ECD kidneys (KDPI < and > 85%) across the eras.

**Figure 8:**
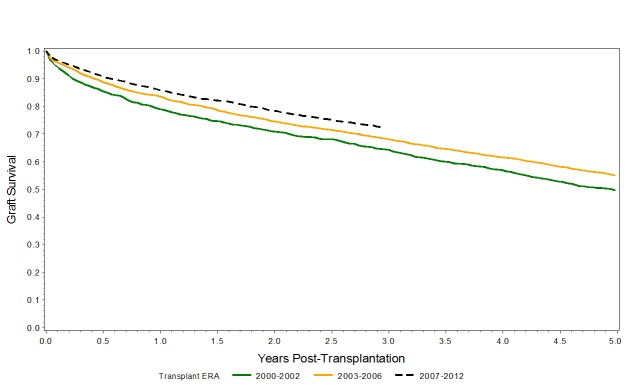
Kaplan-Meier graft survival curves for KDPI >85% Kaplan-Meier curve demonstrating improvement in graft survival across the eras among allografts with KDPI >85%.

When graft survival is plotted with unadjusted Kaplan-Meier graft survival curves, a significant improvement in graft survival across the three eras is observed for the entire cohort (i.e. ECD and KDPI < and > 85%),as shown in Figure 7, as well as for KDPI >85% (p<0.001), as shown in Figure 8*.*

The one-, three- and five-year graft survival rates have also increased across the three eras with the use of kidneys with KDPI >85% (Table 4).

**Table 4:** One-, three-, and five-year graft survival rates across the three eras For kidneys with KDPI >85%, the one-, three-, and five-year graft survival rates have increased across the three eras.

Graft Survival	Era One: 2000-2002		Era Two: 2003-2006		Era Three: 2007-2012	
Time Post Transplant	KDPI ≤85%	KDPI >85%	KDPI ≤85%	KDPI >85%	KDPI ≤85%	KDPI >85%
One month	97.1	94.7	97.6	96.2	98.3	96.9
Six months	93.5	85.5	94.4	88.9	95.7	90.9
One year	90.6	79.1	91.6	83.5	93.5	86
Three years	80.2	64.4	81.8	68.3	84.4	72.2
Five years	69.3	49.8	71.9	55.1	72.3	56.1

However, as expected, when comparing the hazards of graft failure by KDPI, ECD kidney with KDPI >85% has a slightly lower risk of graft failure compared to SCD kidney with KDPI >85%, with a HR of 0.95, probably related to the possibility that some SCD kidneys with a lower estimated quality (higher KDRI) have poor outcomes than some ECDs (Table 5). For KDPI <85%, ECD kidneys have a higher risk of graft failure when compared to SCD.

**Table 5:** Hazards of graft failure by ECD, SCD, and KDPI classification For KDPI <85%, ECD kidneys have a higher risk of graft failure when compared to SCD (HR of 1.51). ECD kidneys with KDPI >85% have a slightly lower risk of graft failure compared to SCD kidneys with KDPI >85% (HR of 0.95).

Donor Type	Comparison	Hazard Ratio
ECD	KDPI: >85% vs. ≤ 85%	1.30
SCD	KDPI: >85% vs. ≤ 85%	2.07
KDPI ≤85%	ECD vs. SCD	1.51
KDPI >85%	ECD vs. SCD	0.95

**Figure 9:**
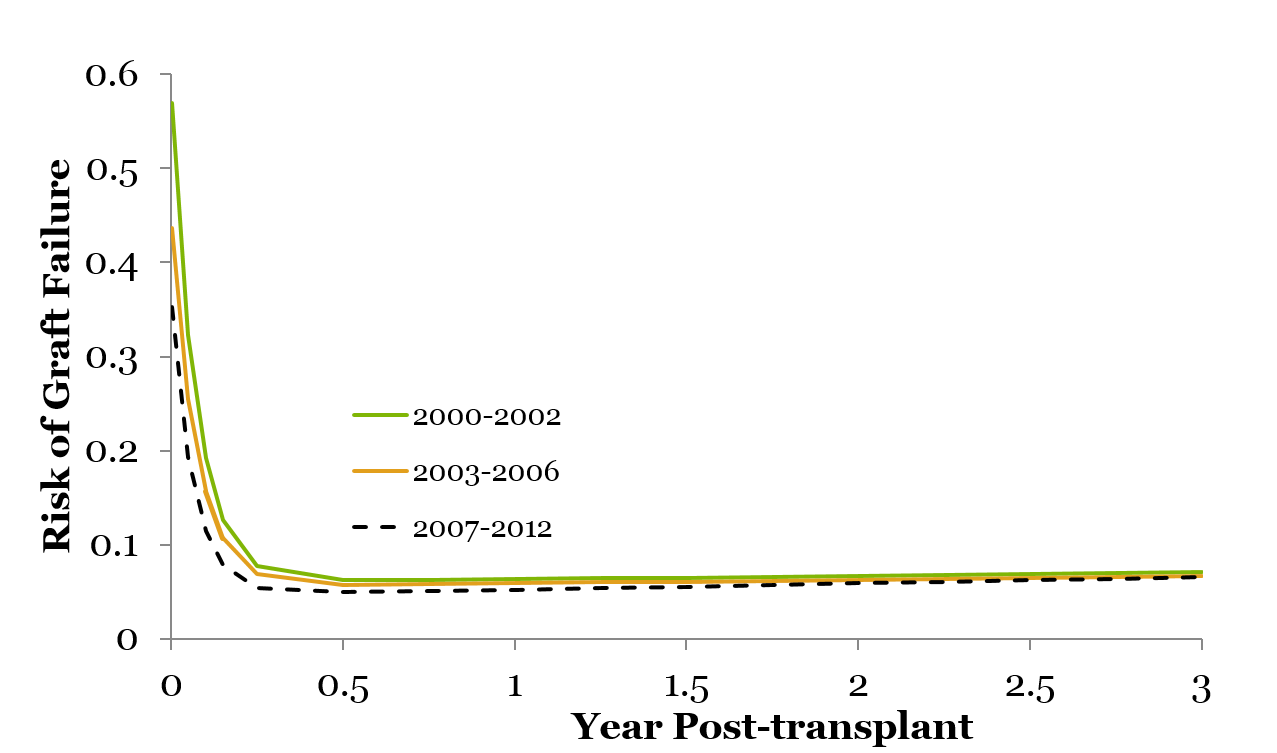
Risk of graft failure for the entire cohort across the eras The modified Bailey-Makeham model for risk of graft failure shows: 1) the initial risk of graft loss (α, the y-intercept) drops across eras; 2) non-significant change in the rates of decline in graft loss (γ, the slope) across eras in approximately the first year; and 3) improvement in the long-term risk over time (βt + δ) in the recent era (2007-2012).

When the risk of graft failure is analyzed using the modified Bailey-Makeham model, on examining the entire cohort together (Figure 9, Table 6), i.e., without any ECD or KDPI characterizations, the observations that come to the forefront indicate that the initial risk of graft loss (alpha, the y-intercept) significantly drops across the eras (α 0.53 vs. 0.39 vs. 0.31; p <0.05). The rates of decline in graft loss (γ, the vertical slope) across the first two eras approximately overlap in the first year; however, some improvement, although not significant, is noted with a faster rate of decline in the most recent cohort (γ 13.82 vs. 13.20 vs. 14.21). The long-term risk of graft loss (βt + δ, the horizontal slope) is significantly improved in the recent era (2007-2012).

**Table 6:** Risk of graft failure from time of transplant, across eras See corresponding Figure 9. The initial risk of graft loss (alpha) significantly drops across eras (α 0.53 vs. 0.39 vs. 0.31; p<0.05). The long-term risk of graft loss (βt + δ) is also significantly improved in Era Three (2007-2012).

	Era One	Era Two	Era Three	P value
Parameter	2000-2002	2003-2006	2007-20012	
Alpha	0.5385	0.3961	0.3101	<0.05 (Ref: 2000-2002)
Gamma	13.8272	13.2022	14.2184	
Beta	0.0038	0.0041	0.0072	<0.05 (Ref: 2000-2002)
Delta	0.0614	0.0568	0.0471	<0.05 (Ref: 2000-2002)

**Figure 10:**
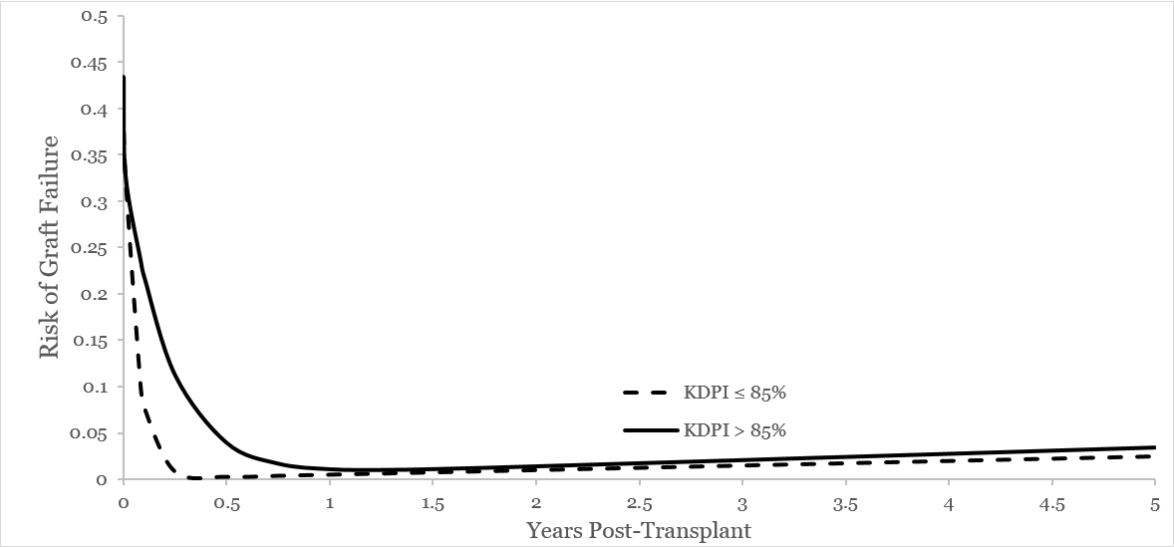
Risk of graft failure characterized by KDPI 85% Modified Bailey-Makeham model for risk of graft failure for kidneys with KDPI >85% demonstrates no difference in initial risk (α, y-intercept) and a slower decline in the risk of graft loss in the first year (γ, the slope) with the risk of graft loss over time accelerating beyond that of lower KDPI organs (βt + δ).

**Table 7:** Risk of graft failure from time of transplant for entire cohort by KDPI See corresponding Figure 10. The initial risk of graft loss (alpha) overlaps between the three eras (α 0.38 vs. α 0.34; p=0.444). KDPI >85% has a significantly slower rate of decline in the risk of graft loss (γ 15.78 vs. γ 4.44; p<0.001) and the long-term risk of graft loss (βt + δ) is higher compared to KDPI <85% organs (δ 0.049 vs. δ 0.089; p<0.001.).

Parameter	KDPI ≤85%	KDPI >85%	P value
Alpha	0.3876	0.3463	0.444
Gamma	15.7836	4.4480	<0.001
Beta	0.0050	0.00697	0.193
Delta	0.0497	0.0895	<0.001

**Figure 11:**
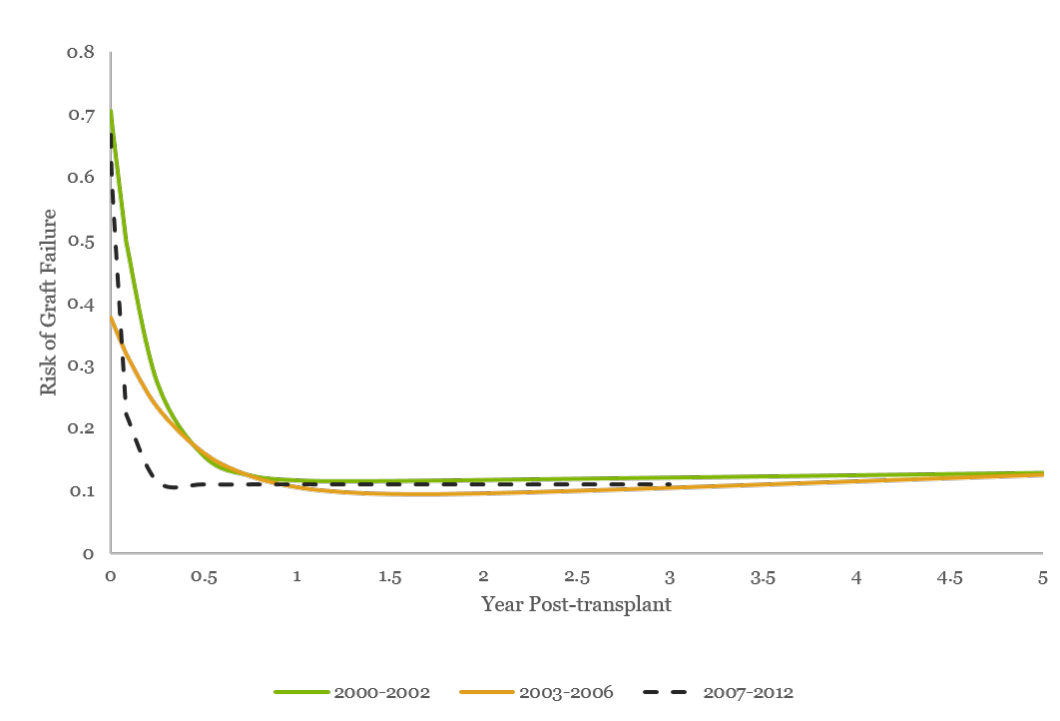
Risk of graft failure for KDPI >85% across the eras Modified Bailey-Makeham model for risk of graft failure from the time of transplant for KDPI >85% across eras: 1) Era Two, 2003-2006, has a lower initial risk of graft loss (α, the y-intercept), while the initial risk is the same for the first and third eras; 2) Era Three, 2007-2012, has a prominent rapid decline in risk of graft failure within the first two months (γ, the slope); and 3) minimal change is seen in the long-term risk of graft loss over the years (βt + δ).

When the risk of graft failure from the time of transplant is characterized exclusively for KDPI >85% across the three eras (Figure 11, Table 8), the observations that come to light include: the initial risk of graft loss α reduces from 0.59 in Era One to 0.30 in Era Two (p < 0.05) but then increases to 0.55 in Era Three, potentially reflecting changes in organ/recipient selection, organ preservation, role of CMS regulation, etc. The rapid decline in risk of graft failure in Era Three (γ 19.43; p<0.05) is most prominent within the first two months, with insignificant changes in this across Eras 1 and 2, once again potentially reflecting changes in recipient matching, organ preservation, and perioperative advances in the most recent era.

**Table 8:** Risk of graft failurefrom time of transplant for KDPI >85% across eras See corresponding Figure 11. Initial risk of graft loss (alpha) reduces from 0.59 in Era One to 0.30 in Era Two (p<0.05). The rapid decline in risk of graft failure in Era Three (γ 5.23 vs. 19.43; p<0.05) is most prominent within the first two months, with insignificant changes across Eras One and Two.

Parameter	Era One: 2000-2002	Era Two: 2003-2006	Era Three: 2007-2012	P value
Alpha	0.596676	0.302393	0.557745787	p<0.05 (Ref: 2000-2002)
Gamma	5.238117	2.630416	19.43761293	p<0.05 (Ref: 2000-2002)
Beta	0.003776	0.010408	5.76951E-06	
Delta	0.108616	0.073145	0.109383634	

Immunosuppression strategies have evolved over the three eras and have impacted the graft survival outcomes in these marginal kidneys. As seen in Table 9, the most modern era has seen a significant increase in the use of lymphocyte-depleting induction agents such as Thymoglobulin-rATG (49.3%) and Campath (14.1%) for kidneys with KDPI >85% (p>0.001). Maintenance immunosuppression (IS) has seen a rise of tacrolimus over cyclosporine across the eras for both KDPI < and > 85%. Use of steroids as maintenance IS has declined over time indicating the use of steroid-sparing regimens probably based on recipient risk profile.

**Table 9:** Immunosuppression strategies across the eras The use of lymphocyte-depleting agents (rATG and Campath) and tacrolimus has increased across the eras, while steriod use has decreased in recent times (p<0.001).

	Era One: 2000-2002		Era Two: 2003-2006		Era Three: 2007-2012		P value
Antibody Induction	KDPI ≤85%	KDPI >85%	KDPI ≤85%	KDPI >85%	KDPI ≤85%	KDPI >85%	KDPI >85%
% rATG	19.1	23.5	40.7	44.6	50.7	49.3	<.0001
% IL2-RA	41.3	38.3	30.8	24.8	24.5	23.6	<.0001
% Campath	-	-	6.9	9.8	11.4	14.1	<.0001
Maintenance IS							
% TAC/% CyA	51.4/39.7	50.2/35.4	72.2/19.2	72.2/14.6	88.7/5.9	87.9/4.8	<.0001
% MPA	76.9	73.6	84.9	83.7	92.7	90.9	<.0001
% Corticosteroids	92.7	89.0	76.1	70.7	67.8	62.7	<.0001

## Discussion

Kidney transplantation utilizing marginal ECD kidneys represents a lifesaving intervention for appropriate candidates [5-6]. The current study outlines the trends and practice patterns in existence related to ECD utilization based on donor organ quality demarcated by the KDPI, as well as lays out the trends in postoperative outcomes following ECD kidney transplantation in the past decade over three distinct eras, using the KPDI tool. Understanding the practice trends for ECD kidneys and analyzing the outcomes with use of such kidneys may help provide an adequate body of evidence and guidance to support decisions in utilizing marginal kidneys as a valuable resource. With further characterization of these trends and outcomes using KDPI definition, additional information can be extrapolated to better understand the utilization of allografts with KDPI >85%. The primary findings of our study indicate that, although the utilization of ECD kidneys initially and subsequently kidneys with KDPI >85% as a percent of total cadaveric kidney transplants has declined in recent years, these organs still constituted a significant percentage of kidneys used between 2000 and 2012. The outcomes following kidney transplantation with KDPI >85% have significantly improved over time, despite worsening donor and recipient risk profiles.

The reduction of initial risk of graft loss across the eras and the rapid decline in the risk of graft failure for ECD kidneys with KDPI >85%, with improvement in the incidence of DGF, not only reflects advances in intraoperative and perioperative management of both the allograft and the recipients, but also indicates improving learning curve with cadaveric kidney transplantation, along with measured performance and quality improvement strategies implemented within transplant centers [17]. This is further supported by the fact that the more recent era has seen a worsening recipient risk profile, which may explain the observation of equivalent, unadjusted patient survival over time, yet better-adjusted survival upon multivariable analysis. This also underscores the improvement in both unadjusted and adjusted graft survival, despite higher-risk recipients. Immunosuppression strategies have evolved over the years and have been modified based on the recipient risk profile and donor kidney quality. Increased use of lymphocyte-depleting agents in the most current era indicates the attempt to minimize calcineurin inhibitor-induced nephrotoxicity to an already marginal organ. Similarly, reduced use of steroids over the era points to the adjustment based on increasing recipient BMI over the eras. However, our data source does not permit definitive conclusions on any “learned” management strategies since information regarding center-specific criteria, intraoperative techniques, fluid and blood product administration, nutrition and pharmacologic management when transplanting kidneys with KDPI >85% are not currently available for analysis. Besides KDPI scores, several other factors such as human leukocyte antigen (HLA) mismatch, age/size mismatch, primary disease recurrence, recipient noncompliance, the presence of donor-specific anti-HLA antibodies, organ damage, or anatomical anomalies which may impact graft outcomes and survival are not taken into account. Further contributions in the literature to delineate most appropriate recipients for these marginal organs, taking into account all given circumstances, may also play a role in improved outcomes.

The etiology for the significant change in the trend of utilization of kidney with KDPI >85% before and after 2008 is multifactorial. Decline in use of such an organ can be related to its association with increased cost and resource utilization compared to a standard criteria kidney [18]. It is interesting to note that timing of the decline in the proportion of ECD kidneys used nationally, temporally coincides with the CMS initiation of Conditions of Participation for organ transplant centers, including the potential exclusion of public funding to centers that do not meet certain performance standards [9]. Concerns have been voiced that CMS actions could result in risk-averse behavior by transplant centers due to the fear of being flagged for poor outcomes, with potentially negative consequences for the field of transplantation with an increase in discard rates of marginal organs [19-20]. Minimizing negative influences of disincentives by appropriate adjustment of performance measures and reimbursement based on donor or recipient risk has been proposed as a method to increase ECD organ utilization [21]. The widespread perception that transplanting marginal kidneys adversely affects overall outcomes, including program-specific reports, elicited discussions through consensus conferences and Ad hoc committees [22] to exclude kidneys with KDPI >85% and consider only low-risk transplants when assessing program performance, thus attempting to reduce center disincentives to transplant high-risk organs. Further studies have also shown that there is no evidence that centers transplanting higher-KDPI kidneys stand at a higher risk for low-performance evaluations, and that risk-aversion behavior does not necessarily benefit program performance but may limit patient access to transplantation [23-24].

Over the years, the decision of utilizing marginal kidneys has become stringent, guided not only by the KDPI score but also by the combined information provided through pretransplant biopsies and data from machine perfusion, which is being used in significantly higher proportions of higher KDPI kidneys. These combined tools have provided some guidance when making organ selection decisions and have potentially contributed to the improved outcomes with use of such kidneys despite decreased utilization over time. However, the usefulness of pretransplant biopsy remains controversial. In relation to this, the United States continues to have a high discard rate compared to European nations, particularly with regard to older donors [25]. In 2011, only 75% of kidneys from registered donors were transplanted, with 734 (9.0%) kidneys discarded from donors where at least one organ was recovered for transplant [1]. With the advent of the KDPI nomenclature, Gandolfini, et al. analyzed the contribution of donor biopsies in the decision of utilization of the so-called marginal kidneys, characterizing them by KDPI [26]. Based on their analysis, kidneys with the highest KDPI may have superior outcomes with a lower Remuzzi score than those with a higher score, indicating some incremental utility for such marginal, ECD, high-KDPI kidneys. Reluctance to use kidneys with KDPI >80% can be obvious from the discard rate of retrieved kidneys between 2002 and 2012 in the USA: 36% of kidneys with KDPI between 80–90% and 63% of kidneys with KDPI >90% were discarded [27]. Pretransplant biopsies evaluated according to the Karpinski-Pirani-Remuzzi score has helped reduce the discard rates of marginal grafts to 15% for kidneys with KDPI of 80–90% and 37% for kidneys with KDPI of 91–100%, indicating that use of KDPI alone as a decision tool in organ allocation can lead to increased discard rates [26].

Replacing the dichotomous SCD/ECD classification system with the KDPI score in order to quantify the risk of graft failure along a continuum has been a topic of debate. To add to this, KDPI has a moderate predictive power (c=0.60) and an inability to differentiate between kidneys of similar KDPI values [9]. While KDPI can serve to encourage the use of marginal kidneys by providing a better risk assessment, a recent study analyzing the impact of providing KDPI with kidney offers concluded that KDPI can result in an increase in the discard rate of discordant SCD kidneys with KDPI >85% through a “labelling effect” [28]. However, KDPI classification also highlights the fact that some SCDs may actually have a lower estimated quality (higher KDRI) than some ECDs. The current study has shown that the hazard risk of graft failure is higher with such discordant SCD kidneys with KDPI >85%. On the other hand, for KDPI <85%, ECD kidneys have a higher risk of graft loss when compared to SCD kidneys. This study also highlights the fact that there is a large variability in the ECD kidneys when categorized by the KDPI. The results of the current study demonstrating improved outcomes with KDPI > 85% kidneys may warrant re-evaluation of an optimal ECD allograft and optimal recipient selection to prevent unnecessary discard of viable organs. Selecting older recipients with lower body mass index and minimizing CIT are some of the practices followed when allocating such marginal organs to optimize the function and reduce the risk of poor outcomes. Using the KDPI categorization, centers are better informed to discuss patients' decisions regarding placement on the KDPI >85% waitlist. UNOS has initiated a pilot study, the Collaborative Innovation and Improvement Network (COIIN), which is a three-year phase I project intended to increase kidney utilization and study new methods of quality monitoring [29]. The 19 transplant centers participating in this study have had success using kidneys that are often declined by transplant programs across the country and will share their practices as well as participate in an alternative, collaborative quality improvement framework to drive improvements in organ offer and acceptance, waitlist management, and care coordination.

The results reported here should be interpreted in the context of the limitations of the study design. This analysis was retrospective in nature; therefore, unmeasured confounders may exist. In addition, data is derived from hundreds of centers, often relying on manual data input and possibility of improper documentation or chances of missing data. Modeling techniques may not entirely account for differences in donor and recipient risk factors when comparing graft and patient survival by year of transplant. Additionally, information regarding discarded ECD kidneys is unavailable in our dataset, precluding further evaluation of causative factors underlying ECD utilization trends. The decision to use the dataset from 2002-2012 was based on its availability at the time of our prior analysis of ECD kidneys. Using the same dataset, we calculated KDPI for each ECD kidney followed by subsequent analysis. Also, using this dataset, there was enough data for observation and reporting one-, three-, and five-year survival outcomes. We do agree that analyzing an additional two years of data using kidneys with KDPI >85% may make more recent experiences available for the utilization of these high KDPI kidneys, considering changing donor age and risk profiles and increased frequency of use of machine perfusion for preservation. Additional studies may also be required to further elucidate on allograft outcomes using SCD kidneys with KDPI >85%.

## Conclusions

Transplantation outcomes using marginal kidneys have improved over time, despite worsening recipient characteristics and donor risk profiles. Despite these findings, the rate of usage of kidneys with KDPI >85% has declined in recent years. The introduction of CMS regulations that penalize centers for poor outcomes has possibly resulted in risk-averse transplant center behavior reflecting an increase in the discard of marginal kidneys with KDPI >85%. This current study analyzes the trends in utilization and outcomes of donor kidneys using KDPI categorization and may offer some understanding and guidance for use of marginal kidneys, thus preserving this valuable resource in the face of an ever-growing population of end-stage renal disease.
